# Beneficial ‘unintended effects’ of a cereal cystatin in transgenic lines of potato, *Solanum tuberosum*

**DOI:** 10.1186/1471-2229-12-198

**Published:** 2012-11-01

**Authors:** Aurélie Munger, Karine Coenen, Line Cantin, Charles Goulet, Louis-Philippe Vaillancourt, Marie-Claire Goulet, Russell Tweddell, Frank Sainsbury, Dominique Michaud

**Affiliations:** 1 Centre de recherche en horticulture, Département de phytologie, Université Laval, Pavillon des Services, 2440 boul. Hochelaga, Québec, QC,, G1V 0A6, Canada; 2 Current address: Horticulture Sciences Department, University of Florida, Gainesville, FL, 32611, USA

**Keywords:** Transgenic crops, Transgene pleiotropy, Unintended effects, Stress/defense-related proteome, Corn cystatin, Potato (*Solanum tuberosum*)

## Abstract

**Background:**

Studies reported unintended pleiotropic effects for a number of pesticidal proteins ectopically expressed in transgenic crops, but the nature and significance of such effects *in planta* remain poorly understood. Here we assessed the effects of corn cystatin II (CCII), a potent inhibitor of C1A cysteine (Cys) proteases considered for insect and pathogen control, on the leaf proteome and pathogen resistance status of potato lines constitutively expressing this protein.

**Results:**

The leaf proteome of lines accumulating CCII at different levels was resolved by 2-dimensional gel electrophoresis and compared with the leaf proteome of a control (parental) line. Out of *ca.* 700 proteins monitored on 2-D gels, 23 were significantly up- or downregulated in CCII-expressing leaves, including 14 proteins detected *de novo* or up-regulated by more than five-fold compared to the control. Most up-regulated proteins were abiotic or biotic stress-responsive proteins, including different secretory peroxidases, wound inducible protease inhibitors and pathogenesis-related proteins. Accordingly, infection of leaf tissues by the fungal necrotroph *Botryris cinerea* was prevented in CCII-expressing plants, despite a null impact of CCII on growth of this pathogen and the absence of extracellular Cys protease targets for the inhibitor.

**Conclusions:**

These data point to the onset of pleiotropic effects altering the leaf proteome in transgenic plants expressing recombinant protease inhibitors. They also show the potential of these proteins as ectopic modulators of stress responses *in planta*, useful to engineer biotic or abiotic stress tolerance in crop plants of economic significance.

## Background

Several studies have described the potential of recombinant protease inhibitors as potent antidigestive compounds to protect crop plants from herbivory or pathogenic infection [1,2]. For instance, cysteine (Cys) protease inhibitors of the cystatin protein superfamily were proposed as protective agents against various herbivorous arthropods and root-parasitic nematodes [3,4]. Cystatins and most other protease inhibitors in plants are competitive protein inhibitors acting as pseudo-substrates to enter the active site of proteases [5]. Following inhibition, the target proteases can no longer cleave peptide bonds, which results in a detrimental inhibition of protein digestive functions in herbivorous pests and accounts for the resistance of several transgenic plant lines expressing recombinant inhibitors.

The heterologous expression of protease inhibitors in plants, however, has raised a number of questions about the possible occurrence of unintended metabolic interference –or pleiotropic effects– on endogenous proteolysis, which could eventually alter important cellular functions [2]. Proteases are ubiquitous metabolic effectors involved in the regulation of numerous cellular processes, ranging from housekeeping functions like protein turnover and the elimination of misfolded polypeptides to the processing of polypeptide pre- and pro-regions on maturing protein backbones [6,7]. While studies have reported negligible phenotypic effects for protease inhibitors in transgenic plants based on the assessment of macroscopic indicators such as growth rate, stem diameter or leaf number [8-10], several reports suggest the onset of more subtle effects at the metabolic level. For instance, plant and mammalian serine (Ser) protease inhibitors ectopically expressed in potato were shown to significantly impact protein levels in leaves, positively or negatively [10,11]. Similarly, recombinant cystatins expressed in Arabidopsis or tobacco were shown to induce a range of phenotype alterations *in planta*, including a delayed development of floral organs [12], a modified physiological behaviour under low temperature or light regimes [13], an altered protein content in leaves [13,14], and a strong repression of the pathogen-inducible hypersensitive response [15].

These findings, along with studies reporting the tolerance of protease inhibitor-expressing plants to abiotic stress cues such as drought, salinity and low temperatures [13,16-18], point to the occurrence of endogenous protease targets for the recombinant inhibitors, directly or indirectly involved in stress-related processes. The so-called pleiotropic effects of recombinant protease inhibitors, which are often considered as unintended metabolic effects in the modified plant, might simply reflect a lack of knowledge on stress-related proteolysis *in planta* and in fact represent a source of potentially useful traits for crop improvement [2]. Here we provide experimental evidence for the up-regulation of abiotic and biotic stress-related proteins in leaves of transgenic potato lines engineered to express corn cystatin II (CCII), a potent inhibitor of C1A Cys proteases exhibiting potential for herbivore pest control [19,20]. We also link the pleiotropic effects of CCII expression with the compromised ability of a model necrotrophic fungus, *Botrytis cinerea*, to colonize leaf tissues of the modified host plant.

## Results and discussion

### Pathogenesis-related proteins are up-regulated in CCII-expressing potato lines

**Figure 1:**
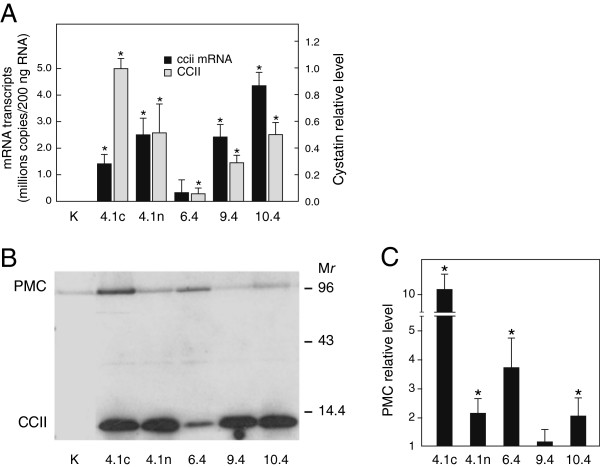
**Expression of CCII and potato multicystatin in leaves of transgenic potato lines. A**. CCII and ccii transcript levels in leaves of control line K and five independent transgenic lines, as determined by SELDI TOF MS and real-time RT PCR. mRNA transcripts were quantified based on a standard curve with incremental amounts of *ccii* transgene as a template. CCII levels are expressed as relative amounts compared to line 4.1c (mean value of 1.0). **B**. Immunodetection of CCII (11.7 kDa) with primary antibodies directed against oryzacystatin I, a structural homologue from rice [22]. Numbers on the right refer to commercial molecular weight markers. PMC refers to endogenous potato multicystatin. **C**. Relative PMC levels in CCII-lines compared to mean basal level in control line K (arbitrary value of 1). Each bar on panels A and C is the mean of three independent (plant replicate) values ± SE. Asterisks indicate a significant difference with control line K (Student’s *t*-test; *P* < 0.05).

Reverse transcriptase (RT) PCR, immunodetection and surface-enhanced laser desorption ionization time-of-flight mass spectrometry (SELDI TOF MS) analyses were performed to select transgenic potato lines expressing CCII at different levels in leaves (Figure 1A,B), among a collection of independent transformants developed earlier in our laboratory [21]. CCII in these lines accumulates in the cytosolic compartment, under the control of the Cauliflower mosaic virus 35S constitutive promoter. Unlike control line K showing no signal, the transgenic lines showed variable, but easily detectable signals of CCII-encoding mRNA transcripts (Figure 1A). Accordingly, CCII was immunodetected as a ~12-kDa polypeptide band of variable intensity in the modified lines, in contrast with line K giving no signal (Figure 1B). The amount of CCII in leaf extracts was variable among the lines, as inferred by SELDI TOF MS after capture of the inhibitor on CM-10 biochips for weak cationic exchange (Figure 1A). Unexpectedly, a clear immunoblot signal was detected in several CCII-lines for the wound inducible homologue of CCII in potato leaves, potato multicystatin (PMC) [22] (Figure 1B). This apparent up-regulation of the endogenous inhibitor was confirmed by densitometric analysis of the immunoblots revealing significant increases reaching two- to tenfold the signals observed for control line K in four transgenic lines, out of five tested (Student’s *t*-test; *P*<0.05) (Figure 1C).

**Figure 2:**
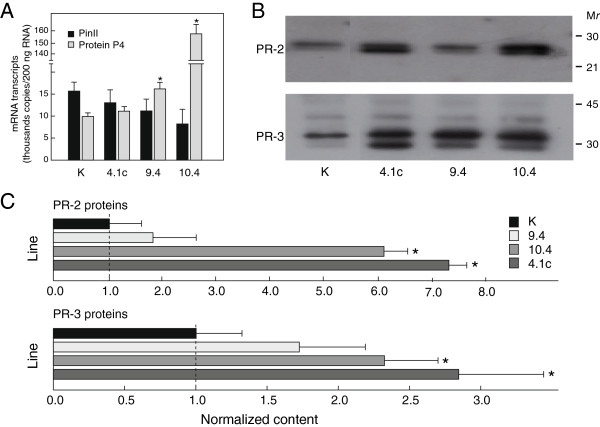
**Expression of endogenous defense proteins in leaves of control line K and CCII-expressing lines 4.1c, 9.4 and 10.4. A**. mRNA transcripts for wound inducible proteinase inhibitor II (Pin-II) and pathogen inducible PR-1 protein P4, as determined by real-time RT PCR. Transcripts were assayed based on standard curves with incremental amounts of *pin-II* or *protein p4* genes as templates. **B**. Immunodetection of PR-2 (β-glucanase) and PR-3 (chitinase) proteins using commercial primary antibodies directed against tobacco structural homologues. Numbers on the right refer to commercial molecular weight markers. **C**. Relative levels of PR-2 and PR-3 proteins in line K (relative value of 1.0) and CCII-lines, as estimated by quantitative densitometry after immunoblot digitalization. Each bar on panels A and C is the mean of three or four independent (plant replicate) values ± SE. Asterisks indicate a significant difference with control line K (Student’s *t*-test; *P* < 0.05).

RT PCR assays were conducted with nucleotide primers for mRNA transcripts of proteinase inhibitor II (Pin-II), a protein marker of wound and jasmonate inducible proteins; and pathogenesis-related (PR) protein P4, a protein marker of the abiotic stress-/pathogen-inducible salicylic acid pathway [23] (Figure 2A). Transcript levels for Pin-II were not different in control and transgenic lines (Student’s *t*-test; *P*=0.10), while transcripts for protein P4 were found at higher levels in several CCII-expressing lines, including line 9.4 and line 10.4 (*P*<0.05). This observation suggesting an up-regulation of salicylate inducible proteins in the CCII-expressing lines was further supported by immunodetection assays with primary antibodies for PR-2 (ß-glucanase) and PR-3 (chitinase) proteins, which showed levels of proteins from both families to be significantly higher in lines 4.1c, 9.4 and 10.4 compared to control line K (Student’s *t*-tests; *P*<0.05) (Figure 2B,C).

### The stress-related proteome is altered in CCII-expressing leaves

**Figure 3:**
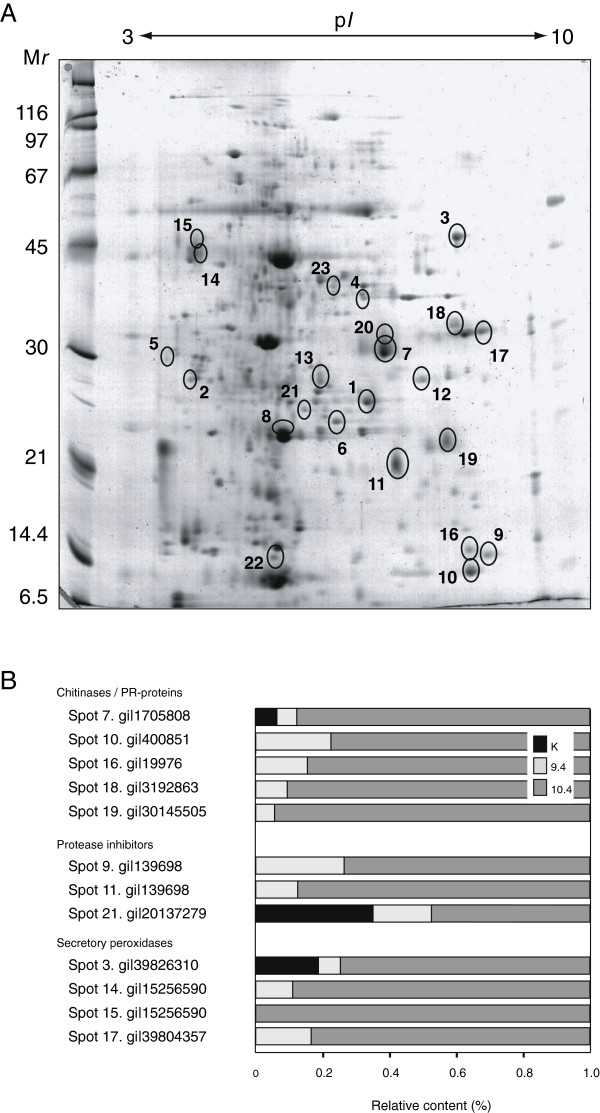
**2-DE analysis of the leaf proteome in control line K and CCII-expressing lines 9.4 and 10.4. A**. Representative 2-D gel image for the leaf proteome of line 10.4, as visualized by colloidal blue staining. A 3 to 10 p*I* gradient was used for isoelectric focusing. Values on the left refer to commercial molecular weight protein markers. Numbered circles refer to proteins up- or downregulated in CCII-expressing lines 9.4 and 10.4, compared to line K (see Table 1 and Additional file 1 for details). **B**. Relative abundance of CCII-inducible stress-related proteins in control line K and transgenic lines 9.4 and 10.4. Data are expressed as relative normalized volumes compared to line 10.4 showing the highest steady-state levels (arbitrary value of 1.0). See panel A and Table 1 for protein numbering and function assignment.

A comparative two-dimensional gel electrophoresis (2-DE) proteome analysis was conducted with leaf protein extracts of line 9.4, line 10.4 and control line K to measure the overall impact of CCII on the host plant’s leaf proteome, and to test the hypothesis of a specific up-regulation of salicylate inducible stress-related proteins in plants ectopically expressing the recombinant inhibitor (Figure 3; Table 1). Out of ~700 protein spots monitored on 2-D gels, 23 showed a significantly altered level in CCII-expressing lines compared to the control line, for similar amounts of protein loaded on gel strips (anova; *P*<0.05) (Figure 3A). Of these proteins, 15 were detected *de novo* or up-regulated by more than fivefold in the CCII-lines (Table 1). As observed above for PR proteins (Figure 2), the up-regulating effects of CCII were systematically stronger in line 10.4 compared to line 9.4 producing lower levels of CCII (Figure 3B), thereby suggesting a dose-dependent effect for the recombinant inhibitor.

**Table 1:** **Proteins up- or downregulated in CCII-expressing potato lines 9.4 and 10.4**^1^

**Spot#**	**Protein identity **^**2**^	**NCBI Accession Number **^**3**^	**Normalized volume **^**4**^		
			**Line K**	**Line 9.4**	**Line 10.4**
1	SOUL heme-binding protein	gi|53779330	0.41	0.40	0.63
2	EGD2 protein	gi|13615277	0.10	0.08	0.04
3	Secretory peroxidase	gi|39826310	0.06	0.08	0.30
4	Mitochondrial NAD-dependent malate dehydrogenase	gi|21388544	0.03	0.05	0.13
6	Multimeric flavodoxin WrbA	gi|537002029	0.02	0.06	0.18
7	Endochitinase 2 precursor	gi|1705808	0.12	0.24	1.93
8	230kDa oxygen evolving protein of photosystem II	gi|11134035	2.90	2.13	1.58
9	WIN1 (wound-induced inhibitor 1) precursor	gi|139698		0.02	0.09
10	Pathogenesis-related protein P2 precursor	gi|400851		0.08	0.36
11	WIN1 (wound-induced inhibitor 1) precursor	gi|139698		0.08	0.64
13	Short-chain dehydrogenase	gi|77403673		0.03	0.22
14	Secretory peroxidase	gi|15256590		0.02	0.21
15	Secretory peroxidase	gi|15256590			0.10
16	Pathogenesis-related protein P2	gi|19976		0.02	0.10
17	Secretory peroxidase	gi|39804357		0.06	0.34
18	1,3ß-glucan glucanhydrolase	gi|3192863		0.02	0.26
19	Osmotin 81	gi|30145505		0.03	0.53
20	Endochitinase 2 precursor	gi|1705808	0.10	0.10	0.01
21	Aspartic protease inhibitor 9	gi|20137279	0.06	0.08	0.16
22	Pentapeptide repeat protein	gi|53782287	0.29	0.20	0.08
23	FBP aldolase Ia	gi|39816882	0.17	0.10	0.04

^1^ 2-DE spatial coordinates, MOWSE score, protein sequence coverage, number of matched peptides and matched peptide sequences are given for each protein in Additional file 1.

^2^ Protein spots 5 and 12 could not be identified based on available MS spectra.

^3^ All protein spots matched potato (*Solanum tuberosum*) protein sequences, except Spot 10 and Spot 16 matching tomato (*Solanum lycopersicum*) sequences.

^4^ Blanks indicate no detectable protein product.

Most proteins up-regulated in the CCII-lines were confidently identified based on LC-MS/MS sequence data (Table 1; Additional file 1). In accordance with the increased levels of PMC and PR proteins in transgenic leaves (see above), several up-regulated proteins in lines 9.4 and 10.4 corresponded to defense-related proteins usually induced upon wounding or biotic stress challenge, such as for instance wound inducible Ser protease inhibitors (Spots 9 and 11 on Figure 3A), a Kunitz cathepsin D inhibitor (Spot 21), a PR-2 (ß-glucanase) protein (Spot 18) and three PR-3 (chitinase) proteins or protein fragments (Spots 7, 10 and 16). Proteins that are usually induced upon abiotic stress challenge and thought to protect cells from oxidative damage or osmotic imbalance were also identified, including an osmotin-like PR-5 protein (Spot 19) and several secretory peroxidases (Spots 3, 14, 15 and 17). A number of studies in recent years have described the potential usefulness of ectopically expressing antioxidant enzymes –or positive regulators of these enzymes– to generate crop lines tolerant to adverse abiotic conditions such as low or high temperatures, drought or salinity [24-28]. Considering the above described pleiotropic effects of CCII on the potato leaf proteome, the reported ability of cystatin-expressing plants to cope more efficiently with abiotic stress conditions [13,16] could be likely explained, at least in part, by a significant up-regulating effect of the recombinant inhibitors on the steady-state levels of these enzymes in leaf tissues.

### CCII does not inhibit the extracellular proteases of *Botrytis cinerea*

Bioassays were conducted with the fungal necrotroph *B. cinerea* to document the possible positive impact of CCII pleiotropic effects, including PR protein up-regulation, on potato plants challenged with an aggressive microbial pathogen. The increased expression of peroxidases and PR proteins in leaves treated with benzathiadiazole, a functional analogue of salicylic acid, was shown earlier to induce resistance to *B. cinerea* in different plants (e.g. [29,30]). Likewise, strong detrimental effects against this pathogen were reported for recombinant PR proteins or gene inducers of these proteins expressed in different plants, including potato [31-34]. The documented repressing effect of recombinant cystatins on the pathogen-inducible hypersensitive response in transgenic plants [15] could also impact the ability of *B. cinerea* to colonize CCII-leaf tissues. This pleiotropic effect of recombinant cystatins*,* also observed in plants treated with synthetic Cys protease inhibitors or transformed with DNA sequences to silence Cys proteases expression [35], would be correlated with an increase of cystatin:Cys protease ratios *in planta* preventing the release of free Cys proteases to initiate cell death [36]. This ectopic phenotype could represent, along with the constitutive over-expression of stress-related proteins, a major hurdle to the progression of the necrotroph, which relies on cell death induction to generate dead tissues and successfully colonize the host plant [37-41].

**Figure 4:**
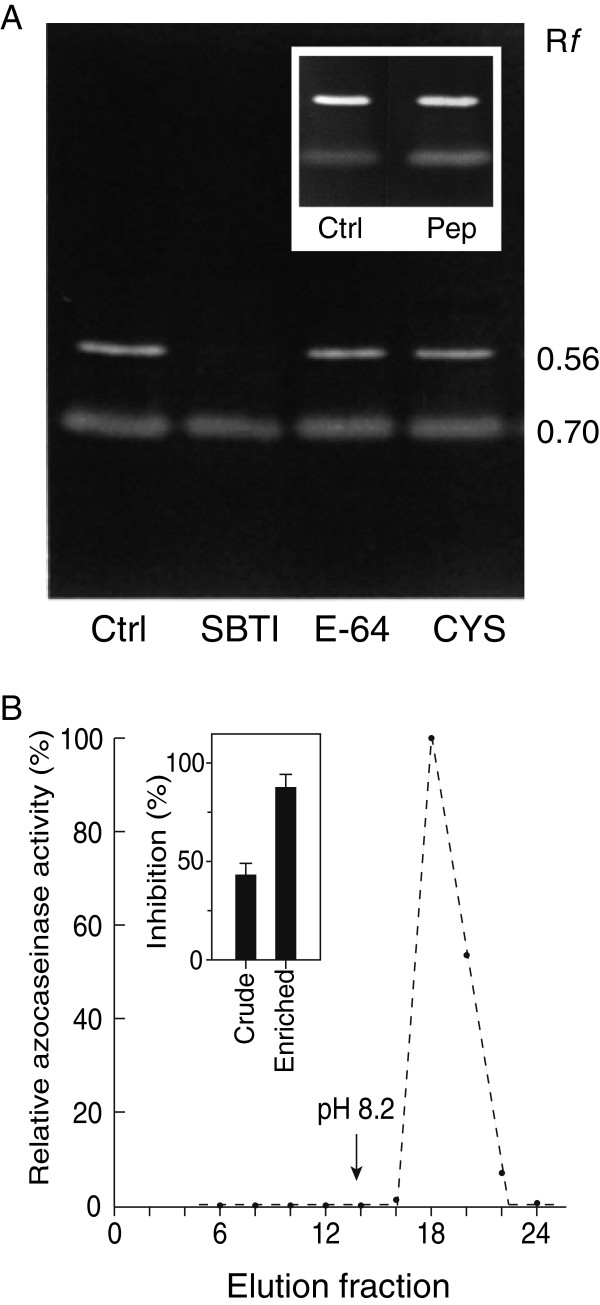
**Secreted proteases of the fungal necrotroph *Botrytis cinerea*. A**. Gelatin/SDS-PAGE zymogram showing two protease (gelatinase) bands, pre-incubated or not with class-specific protease inhibitors before electrophoretic separation. Ctrl, control sample, with no inhibitor added; SBTI, soybean trypsin inhibitor; E-64, *trans*-epoxysuccinyl-L-leucylamido-(4-guanidino) butane; CYS, oryzacystatin. R*f* values on the right refer to relative mobilities in the gel, compared to the protein stain front (arbitrary value of 1.00). **B**. Elution pattern of *B. cinerea* secreted Asp proteases affinity-purified with pepstatin-agarose beads. The protein extract was passed through a 3-ml pepstatin A-agarose column, and the bound proteases eluted by pH increase at 8.2. The elution fractions were monitored for azocaseinase activity and sensitivity to pepstatin inhibition (see text for details). The relative inhibitory effects of pepstatin on crude (non-purified) and pepstatin-enriched samples are shown in the inset graph. Data are expressed as relative inhibitory activities compared to a non-inhibited control (0% inhibition). Each bar is the mean of three independent values ± SE.

Protease activity assays were first performed to measure the relative abundance of secreted Cys proteases in *B. cinerea*, and to evaluate the possibility of a direct, Cys protease inhibitory-mediated effect of CCII against this fungus. In agreement with earlier studies reporting the presence of aspartate (Asp) proteases in the secretome of *B. cinerea*[42,43] and proposing a key role for these enzymes during plant infection [44-46], protease (azocaseinase) activities in the liquid phase of a *B. cinerea* culture grown in potato dextrose broth were inactivated by almost 50% with pepstatin A, a specific inhibitor of A1 Asp proteases (Table 2). Ser protease activities were also detected, as inferred by a weak, but reproducible inhibitory effect of Ser-type inhibitors on azocaseinase activity. The presence of (a) secreted Ser protease(s) in the culture medium was confirmed by the detection of a clear protein lysis band following gelatin/SDS-PAGE, strongly inhibited by the Ser-type inhibitor soybean trypsin inhibitor (Figure 4A, R*f* mobility of 0.56). A second band was detected in gelatin-polyacrylamide gels, with an R*f* mobility of 0.70. This protease, mostly active in acidic conditions (not shown) and insensitive to commonly used class-specific diagnostic inhibitors including pepstatin A (Figure 4A, inset gel), likely corresponded to the pepstatin-insensitive G1 Asp protease BcACP1 identified as an additional determinant of pathogenic infection by *B. cinerea*[46]. Pepstatin-sensitive azocaseinases could not be visualized following gelatin/PAGE as reported earlier for other A1 Asp proteases [47], but could be enriched in mild conditions by affinity chromatography with pepstatin-agarose beads (Figure 4B). Unlike Asp and Ser protease inhibitors, Cys-type inhibitors, such as the fungal inhibitor *trans*-epoxysuccinyl-l-leucylamido-(4-guanidino) butane (E-64) and different cystatins of plant or animal origin, did not inhibit azocaseinase or gelatinase activities (Table 2; Figure 4A). Overall, these observations pointed to the net predominance of Asp and Ser proteases in the culture medium of *B. cinerea*, as suggested by recent high-throughput proteomic studies on the secretome of this model organism [42,43]. They also suggested the absence of Cys protease targets for cystatins and the likely innocuity of CCII against the fungus.

**Table 2:** **Inhibitory effect of diagnostic protease inhibitors on *Botrytis cinerea *secreted proteases **^**1**^

**Inhibitor **^**2**^	**Target proteases**	**Working concentration (μM)**	**Inhibitory activity (%)**
Pepstatin A	Asp	30	46 ± 3
PMSF	Ser	3,000	15 ± 2
AEBSF	Ser	300	7 ± 2
Soybean trypsin inhibitor	Ser	10	7 ± 2
Leupeptin	Cys/Ser	300	2 ± 2
E-64	Cys	100	3 ± 1
Chicken egg white cystatin	Cys	25	3 ± 4
Oryzacystatin I	Cys	25	3 ± 3
Oryzacystatin II	Cys	25	2 ± 3
EDTA	Metallo-	3,000	3 ± 3

^1^An azocaseinase assay was performed at pH 5.0, for 3 h at 37°C. Values are expressed as relative inhibitory activities compared to an uninhibited control (0% inhibition). Each datum is the mean of three values ± SE.

^2^AEBSF, 4-(2-aminoethyl)-benzenesulfonyl fluoride, hydrochloride; E-64, *trans*-epoxysuccinyl-l-leucylamido-(4-guanidino) butane; EDTA, ethylenediamine tetraacetic acid; PMSF, phenylmethylsulfonyl fluoride.

### The pleiotropic effects of CCII induce resistance to *Botrytis cinerea* in potato

**Figure 5:**
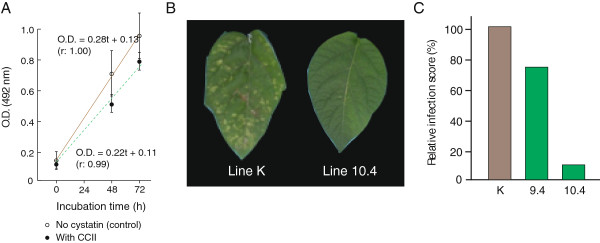
**Impact of recombinant CCII and CCII-expressing potato lines on growth and infection capacity of *Botrytis cinerea*. A**. Fungal growth in potato dextrose broth, in the presence or absence of purified CCII [20]. Optical density of the fungal culture at 492 nm was used as an indicator of biomass production [53]. Datapoints are the mean of three independent (biological replicate) values ± SE. **B**. Typical disease symptoms observed on *B. cinerea*-inoculated plants of line 10.4 and control line K, 12 days post-infection. **C**. Relative disease symptom scores after 12 days for line 9.4, line 10.4 and control line K plants inoculated with *B. cinerea* conidia. Data are expressed as relative cumulative symptom scores, compared to line K (100%) (see Methods).

Because studies have reported negative effects for plant cystatins against a number of pathogenic fungi including *B. cinerea* (e.g. [48-52]), *in vitro* bioassays were carried out with purified CCII to rule out a possible, although unexpected, protease inhibition-independent toxic effect against the model fungus. In contrast with studies reporting significant effects for barley and strawberry cystatins [51,52], CCII had no significant effect on fungal biomass production (Student’s *t*-test; *P*=0.18) (Figure 5A). This observation, along with both the retention of CCII in the cytosol of potato leaf cells (see [21]) and the absence of Cys protease targets in the extracellular milieu (above), confirmed the relevance of *B. cinerea* as a pathogenic model to assess the impact of CCII-mediated, defense-related pleiotropic effects in the CCII-expressing lines, without the risk of confounding effects due to fungal protease inhibition.

*In vivo* bioassays were conducted with a conidial suspension of the pathogen to compare leaf infection dynamics and symptom development patterns on leaves of line 9.4, line 10.4 and control line K (Figure 5B,C). Visual symptoms were recorded over 12 days post-infection and categorized along a ‘0 to 5’ scale, where 0 indicated no symptom and 5 visible mycelium growth at the leaf surface (see Methods). As expected, line K exhibited several symptoms, starting with chlorosis of mesophyll tissue after a few days, followed by tissue necrosis at the inoculation site, black areas at leaf extremities indicating vascular tissue infection, and silver spots on most leaves indicative of active mycelium growth. By comparison, no fungal mycelium could be observed on the CCII-expressing leaves, and necrosis symptoms were systematically less severe, especially for line 10.4 expressing larger amounts of CCII (Figure 5B). The incidence rate of visual symptoms after 12 days was significantly lower on transgenic leaves (anova; *P*<0.01), as inferred from a relative cumulative symptom score of less than 10% for line 10.4 compared to control line K (Figure 5C). These observations pointed overall to a protective effect of CCII in potato plants interacting with *B. cinerea*, likely explained by the stress-related pleiotropic effects of this inhibitor in leaf tissues.

## Conclusion

Studies have reported significant pleiotropic effects for recombinant cystatins expressed in transgenic plant lines, notably including stress tolerance phenotypes suggesting an impact on endogenous stress-related responses. In line with these findings, our data showing an up-regulation of abiotic and biotic stress-related proteins in CCII-expressing potato leaves point to the onset of cystatin-mediated pleiotropic effects *in planta* altering at some degree the host plant stress metabolism. In particular, our proteomic data suggest a protein-inducing impact of recombinant CCII in potato leaves triggering the constitutive expression of normally inducible defense proteins, and the beneficial impact of this (still unintended) phenotype on plants challenged with the fungal pathogen *B. cinerea*.

The exact metabolic events driving the up-regulation of stress-related proteins in CCII-leaves remain unclear at this stage. The constitutive expression of several proteins naturally induced by salicylate or its functional analogue benzathiadiazole suggests a role for the salicylic acid signaling pathway, but a more complex picture involving alternative defense pathways cannot be ruled out. For instance, multicystatins in *Solanum* species are induced by wounding or jasmonic acid [22] but do not respond to fungal elicitors known to reproduce the inducing effects of salicylic acid [54], in apparent disagreement with the hypothetical up-regulation of PMC in CCII-potato lines via a salicylate inducible route. In a similar way, the actual effects of salicylate inducible defenses against *B. cinerea* remain equivocal, given the contrasting effects observed from one plant to another [55] and the various signaling interactions established between host plants and different strains of the pathogen [56]. The high complexity of host plant–*B. cinerea* interactions *in vivo* was further illustrated, recently, by a study reporting the deliberate induction of salicylate inducible responses in *Nicotiana benthamiana*, as a strategy for the pathogen to avoid harmful effects of jasmonate inducible defenses [57].

From a biochemical viewpoint, the occurrence of jasmonate and salicylate inducible proteins in CCII-leaf cells suggests, at this point, the onset of gene (protein)-specific effects for the cystatin, presumably initiated by the inhibition of one or several C1A Cys proteases in the cytosolic compartment. Work is underway to identify such putative protease targets, by a dual *in silico*/empirical approach involving bioinformatic inferences from Solanaceae genome databases and formal detection of cystatin:protease complexes using activity-based, functional proteomics strategies. Work is also underway to modulate cystatin:Cys protease interactions and their resulting pleiotropic effects *in planta*, using cystatin single variants engineered to exhibit either stronger or weaker inhibitory activity against Solanaceae protease targets [58,59]. The proteomic data provided here should represent, to this end, a useful tool for the efficient, one-step monitoring of multiple stress-related protein alterations in plants expressing the recombinant inhibitors.

## Methods

### CCII-expressing potato lines

Transgenic potato lines expressing CCII were selected from a collection of independent transformants derived from *Solanum tuberosum* plantlets, cv. Kennebec (see ref. [21] for details on transgene construct). Line K used as parental line for genetic transformation was selected as a control for comparative purposes [9]. The plantlets were acclimated and grown in greenhouse under a 16h:8h light-day photoperiod. Leaf samples for biochemical analyses were frozen in liquid nitrogen and stored at −80°C until use.

### CCII detection and quantitation

mRNA transcripts for CCII in transgenic potato lines were isolated from the 4^th^ leaf of 30-cm plants and assayed by real-time RT PCR as previously described [21], using the following sense and antisense oligonucleotide primers: 5’–GGACGACCTTCACCTCCAGGAGCTC–3’ and 5’–GTACATCGTGCCAGCAACCACTTGTG–3’. CCII was immunodetected in soluble protein extracts of the 4^th^ leaf using polyclonal primary antibodies raised in rabbits against oryzacystatin [21]. Relative CCII levels in leaf extracts were assayed by SELDI TOF MS using the Ciphergen’s PBSIIc ProteinChip Reader for sample processing, and the Ciphergen ProteinChip software, v. 3.2.0, for data collection and analysis (Bio-Rad, Mississauga ON, Canada) (see [60] for protocol details). CM10 protein biochips for weak cationic exchange were used for protein capture prior to MS analysis. RT PCR and SELDI assays both involved three biological (plant) replicates to allow for statistical assessments.

### Expression of defense-related proteins

mRNA transcripts for the stress protein markers Pin-II and protein P4 were isolated from the 4^th^ leaf of 30-cm plants and assayed by real-time RT PCR as described [21], using the following sense and antisense DNA primers: 5^′^–GCGAAGGCTTGCACTTTAGAATGTG–3^′^ and 5^′^–GGACAAGTCTAGGGTCACATTGCAGGGTAC–3^′^ for Pin-II; and 5’–CTCACTTGTCTCATGGTATTAGCC–3^′^ and 5^′^–CAGGATCATAGTTGCATGAAATG–3’ for protein P4. PR-2 proteins (ß-glucanases) and PR-3 proteins (chitinases) were immunodetected as previously described [21], using polyclonal primary antibodies raised in rabbits against tobacco PR-2 or PR-3 proteins (AgriSera, Vännäs, Sweden). Densitometric analysis of the PR protein signals was performed with the Phoretix 2D Expression software, v. 2005 (NonLinear USA, Durham NC, USA), after scanning the immunoblots with an Amersham Image Scanner digitalizer (GE Healthcare, Baie d’Urfé QC, Canada). PCR and immunodetection assays involved three or four independent (biological) replicates to allow for statistical assessments.

### Protein extraction for 2-DE

Proteins for 2-DE were extracted from the 4^th^ leaf of 30-cm plants. Leaf tissues were ground in liquid nitrogen, and the proteins precipitated at −20°C for 12 h in 10% (v/v) trichloroacetic acid diluted in acetone. Insoluble material was recovered by centrifugation at 4°C for 25 min at 16,000 *g*, and Speed-Vac centrifugation for 5 min at 20°C. The proteins were resolubilized in an electrophoretic sample buffer consisting of 8 M urea (Sigma-Aldrich, Oakville ON, Canada) with 2% (w/v) CHAPS, 0.5% (v/v) IPG buffer 3–10 (GE Healthcare) and 9.3 mg ml^-1^ dithiothreitol (Sigma-Aldrich). Protein concentrations were assayed according to Bradford [61], with bovine serum albumin as a standard.

### 2-DE

Isoelectric focusing (IEF) for 2-DE was performed along a 3 to 10 non-linear pH gradient in 13-cm Immobiline DryStrip gel strips (GE Healthcare), with 200 μg of leaf protein diluted in 250 μl of electrophoretic sample buffer (see above). Proteins were applied to the strips and resolved using an IPGphor system (GE Healthcare). The running program for IEF involved the following steps: rehydration for 12 h at 30 V; 100 V for 1 h; 500 V for 1 h; 1,000 V for 1 h; 5,000 V for 1 h; and 8,000 V to reach 19,000 Vh. After IEF, the strips were incubated for 15 min in 50 mM Tris–HCl equilibration buffer, pH 8.8, containing 6 M urea, 30% (v/v) glycerol, 2% (w/v) SDS and 10 mg ml^-1^ dithiothreitol, and used immediately for the second dimension. Standard 12% (w/v) SDS-PAGE [62] was performed in 1 mm-thick polyacrylamide slab gels. After migration, the gels were fixed overnight in water containing 10% (v/v) acetic acid and 40% (v/v) methanol, and then washed three times in water. Proteins were stained with the GelCode reagent (Pierce, Rockford IL, USA), and the gels scanned using an Amersham Image Scanner digitalizer prior to computer processing.

### Image analysis and protein identification

Image analysis of the 2-D gels was carried out using the Phoretix 2D Expression software, v. 2005 [10], with three biological (plant) replicates for each treatment to allow for statistical analyses (anova; α=0.05, *q*=0.05). Protein spots selected for identification were manually excised from the gels, and subject to trypsin automated in-gel digestion in a MassPrep Workstation (Micromass, Manchester UK) [63]. LC-MS/MS analysis was performed at the Génome Québec Centre of McGill University (Montréal QC, Canada), using a Q-TOF micro apparatus (Waters, Milford MA, USA) and a nanosource modified with a nanospray adapter (New Objective, Woburn MA, USA) to hold a PicoFrit column (BioBasic C18 packing, 5 μm, 300 Å). Peptide sequence data were searched against the NCBInr and NCBI Viridiplantae EST databases, using the Mascot algorithm for protein identification ( http://www.matrixscience.com) (Matrix Science, London, UK). Search parameters for protein matching were as follows: one trypsin miscleavage allowed, peptide mass tolerance of 0.3 Da, carbamidomethylated Cys residues (fixed modification) and Met residues in oxidized form (variable modification). Protein identifications were accepted when matching scores were significant at α=0.05, based on the MOWSE score (Matrix Science).

### *Botrytis cinerea* extracellular proteases

Extracellular proteases of *B. cinerea*, isolate 8–8170, were recovered from the liquid phase of a 250-ml culture grown in potato dextrose broth (Difco, Burlington ON, Canada). The fungus was allowed to grow for two weeks in the dark at 20°C. Fungal mycelium was discarded by filtration through a 0.2 μm filter, and the extracellular proteins concentrated by ammonium sulfate precipitation at 90% (w/v) saturation. The mixture was centrifuged for 20 min at 13,000 *g*, the proteins resuspended in 100 mM citrate phosphate, pH 6.0, and the resulting solution desalted by gel filtration in a Sephadex G-25 column (GE Healthcare) using the same buffer. Total protease (azocaseinase) activity was measured in mild reducing conditions at pH 6.0, in the presence or absence of class-specific protease inhibitors (see [64]). The proteases were also resolved by gelatin/SDS-PAGE in non-reducing conditions, after pre-incubating the extracts for 15 min with class-specific inhibitors [65]. An Asp protease not detected in gelatin-containing gels was enriched by pepstatin A affinity chromatography, using pepstatin-agarose beads (Sigma-Aldrich) pre-equilibrated with 100 mM citrate acetate, pH 5.0, containing 1 M NaCl [66]. The samples were passed through a 3-ml agarose column, washed with several volumes of pre-equilibration buffer, and eluted in basic conditions with 100 mM Tris–HCl, pH 8.2. Fifty-μl fractions were collected in eppendorf tubes containing 50 μl of 100 mM sodium acetate buffer, pH 3.5, and used as source material for azocaseinase assays (see above).

### Impact of recombinant CCII on *Botrytis cinerea* growth

The impact of CCII on growth of *B. cinerea* was assessed by monitoring the optical density of fungal liquid cultures containing a bacterially produced form of the inhibitor [20] at 10 μM final concentration. The fungus was allowed to grow in the dark at 20°C in potato dextrose broth (Difco). The optical density at 492 nm, indicative of fungal biomass production *in vitro*[53], was recorded 0, 48 and 72 h after culture initiation, and compared with the corresponding density of a control culture grown in the absence of CCII. Three biological replicates were used for the assay to allow for mean comparisons (Student’s *t*-test; α=0.05).

### Impact of CCII-expressing potato lines on *Botrytis cinerea* infection

The impact of CCII expression on host plant’s resistance to *B. cinerea* was assessed by comparing the incidence and severity of necrotic areas on leaves of control and CCII-expressing lines infected with conidial suspensions of the pathogen. A solid plate culture of the fungus established on potato dextrose agar (Difco) was incubated for three days at 17°C, under a 12-h UV light daily regime to induce sporulation. The conidia were washed for 10 min in 10 ml of water containing 0.05% (v/v) Tween 80, counted with a hemocytometer, and their concentration adjusted to 4 × 10^5^ conidia ml^-1^. The leaves of seven weeks-old control and CCII-expressing plants were wounded with a scrub sponge to weaken the cuticle, and infected with 30 μl-droplets of the conidial suspension. Relative humidity in the greenhouse was raised to 90% for 72 h, and then reduced to 70% for 12 days, before recording disease incidence. Visual symptoms were classified along a ‘0 to 5’ scale, with a score of 0 indicating the absence of disease symptom. A score of 1 was characterized by the presence of black spots on the vascular tissues; a score of 2 by the presence of chlorosis and necrotic areas; a score of 3 by necrotic lesions covering up to 30-50% of the leaf surface; a score of 4 by necrotic lesions covering more than 50% of the leaf surface; and a score of 5 by the evidence of mycelium growth. Four plant replicates were used for each line, with seven leaves (i.e. leaf 3 to leaf 9, from the apex) inoculated and monitored on each plant to avoid potential confounding effects of leaf age-dependent transgene expression in potato leaves (see [67]). Statistical comparisons of mean values were performed following a logarithmic transformation of the raw data (anova; α=0.05). Cumulative symptom scores were calculated for each plant line replicate, and the mean cumulative score for each transgenic line compared to the mean cumulative score of control line K (100%).

## Abbreviations

2-DE: Two-dimensional gel electrophoresis; CCII: Corn cystatin II; Asp: Aspartate; Cys: Cysteine; E-64: *trans*-epoxysuccinyl-l-leucylamido-(4-guanidino) butane; IEF: Isoelectric focusing; PMC: Potato multicystatin; PR-2 (or PR-3) proteins: Pathogenesis-related proteins 2 (or 3); RT PCR: Reverse transcriptase PCR; SELDI TOF MS: Surface-enhanced laser desorption ionization time-of-flight mass spectrometry; Ser: Serine.

## Competing interests

The authors declare that they have no competing interests.

## Authors’ contributions

AM contributed to the experimental design, performed the experiments, and wrote a first draft of the manuscript. KC, LC and LPV contributed to the experiments and to data analysis. CG, RT, MCG and FS contributed to the experimental design and writing of the manuscript. DM conceived the study, contributed to the experimental design, coordinated the experiments, and prepared the last version of the manuscript. All authors read and approved the final manuscript.

## Supplementary Material

Additional file 1 LC-MS/MS identification of leaf proteins up- or downregulated in CCII-expressing potato lines 9.4 and 10.4. Click here for file
