# Supplementary material for: Beneficial ‘unintended effects’ of a cereal cystatin in transgenic lines of potato, Solanum tuberosum
Source: BMC Plant Biol. 2012 Nov 1;12:198. doi: 10.1186/1471-2229-12-198 (PMC3534561; doi:10.1186/1471-2229-12-198)
Supplement: Additional file 1 — LC-MS/MS identification of leaf proteins up- or downregulated in CCII-expressing potato lines 9.4 and 10.4. [file 1471-2229-12-198-S1.pdf]

Additional file 1: LC-MS/MS identification of leaf proteins up- or downregulated in CCII-expressing potato lines 9.4 and 10.4

Spot	Identification	Accession number[1]	MOWSE score	p*I* [2,3] (Exp./Calc.)	M_r [2,3] (Exp./Calc.)	No. peptides	% Coverage	Matched unique peptides
1	SOUL heme-binding protein	gi|53779330	246	6.7/9.0	25.9/31.0	4	17	R.LFDYIQGK.N<br>K.NQPNPPPAEGLHLQK.W<br>R.QFSGFITDDDLPR.E<br>R.EAAALSASIAGTK.W
2	EGD2 protein	gi|13615277	94	3.5/5.6	29.0/16.9	3	25	K.DIELVMTQAGVSR.A<br>K.IEDLSSQLQSQAAEQFK.A<br>K.APNLSNVISKPEPSTVAQDDEDVDET GVEPK.D
3	Secretory peroxidase	gi|39826310	376	9.0/8.5	45.0/35.6	6	23	R.RLQNVFR.Q<br>R.QDIGQAAGLLR.L<br>R.VVSCADITAIAAR.D<br>R.DSVFFSGGPDYDLPLGR.R<br>R.LYPNQDSSMDK.T<br>K.MGQLNVLTGTQGQIR.G
4	Mitochondrial NAD-dependent malate dehydrogenase	gi|21388544	282	6.6/8.9	35.0/36.4	5	18	R.SEVVGFAGEEQLGK.A<br>K.ALEGADIVIIPAGVPR.K<br>R.DDLFNINAGIVK.S<br>K.LFGVTMDLVVR.A<br>R.TQDGGTEVVEAK.A
5	Unknown protein	gi|51457942	33	3.2/4.8	29.8/25.0	1	---	K.VSMEWVAMDNTK.V
6	Multimeric flavodoxin WrbA	gi|537002029	381	6.3/6.7	24.0/28.0	5	20	K.KGAASVEGVEAK.L<br>K.GAASVEGVEAK.L<br>K.LWQVPETLSEEVLGK.M<br>R.FGMMAAQFK.A<br>K.GGSPYGAGTFAGDGSR.Q
7	Endochitinase 2 precursor	gi|1705808	126	6.9/5.9	30.0/34.6	4	22	R.WQPSGTDQAANR.V<br>R.GPIQISHNYNYGPCGR.A<br>R.YCGILGVSPGDNLDCGNQR.S<br>R.VPGFGVITNIINGGLECGHGSDSR.V

Additional file 1: LC-MS/MS identification of leaf proteins up- or downregulated in CCII-expressing potato lines 9.4 and 10.4 (Cont'd)

Spot	Identification	Accession number[1]	MOWSE score	p*I* [2,3] (Exp./Calc.)	M_r [2,3] (Exp./Calc.)	No. peptides	% Coverage	Matched unique peptides
8	23 kDa subunit of oxygen evolving protein of photosystem II	gi|1771778	337	5.2/8.3	22.7/28.2	7	28	K.QAYFGK.T<br>K.VDYLLGK.Q<br>K.EIEFPGQVLR.Y<br>K.HQLITATVNDGK.L<br>K.QYYYLSVLTR.T<br>K.SITDYGSPEEFLSK.V<br>K.ENTDFLPYNGDGFK.L
9	WIN1 (wound-induced inhibitor 1) precursor	gi|139698	54	9.4/8.6	15.1/22.2	1	4	R.TGAQTTVR.I
10	Pathogenesis-related protein P2 precursor	gi|400851	118	9.2/8.5	14.0/16.4	2	16	R.LDTNGLGYQR.G<br>R.VTNTGTGTQETVR.I
11	WIN1 (wound-induced inhibitor 1) precursor	gi|139698	56	7.2/8.6	21.1/22.2	1	4	R.TGAQTTVR.I
12	---	---	---	8.0/---	28.2/---	---	---	---
13	Short-chain dehydrogenase	gi|77403673	182	6.1/6.2	28.5/27.2	3	12	R.VAIVTGSSR.G<br>K.ANVSDPDQVK.S<br>K.YPSILNTPLEDFDR.T
14	Secretory peroxidase	gi|15256590	75	3.6/6.3	44.8/25.6	2	15	K.DAAANVGAGGFDIVDDIK.T<br>K.GMDITDLVALSGAHTFGR.A
15	Secretory peroxidase	gi|15256590	131	3.5/6.3	45.2/25.6	3	25	K.DAAANVGAGGFDIVDDIK.T<br>K.GMDITDLVALSGAHTFGR.A<br>R.TGVDTDIPSPVESLDVMRPQFTNK.G
16	Pathogenesis-related protein P2	gi|19976	45	9.2/8.5	15.3/16.4	1	9	R.VTNTGTGTQETVR.I
17	Secretory peroxidase	gi|39804357	171	9.2/7.7	32.4/33.0	5	18	R.GILESDQK.L<br>R.GYEVIDDAK.Q<br>R.AESIVQSTVR.S<br>R.VGFYSSTCPR.A<br>R.ASDASNLPGFTESVDAQK.Q

Additional file 1: LC-MS/MS identification of leaf proteins up- or downregulated in CCII-expressing potato lines 9.4 and 10.4 (Cont'd)

Spot	Identification	Accession number[1]	MOWSE score	p*I* [2,3] (Exp./Calc.)	M_r [2,3] (Exp./Calc.)	No. peptides	% Coverage	Matched unique peptides
18	1,3ß-glucan glucanhydrolase precursor	gi|3192863	122	8.9/6.7	33.0/39.9	3	12	K.AIGEAGLGNDIK.V<br>R.WFTDPIVGFLR.D<br>K.VSTSVDMTLIGNSYPPSQGSFR.N
19	Osmotin 81	gi|30145505	119	8.7/8.4	23.0/19.9	2	11	R.GQTWVINAPR.G<br>R.TNCNFDGAGR.G
20	Endochitinase 2 precursor	gi|1705808	38	6.9/5.9	31.0/34.6	2	22	R.WQPSGTDQAANR.V<br>R.GPIQISHNYNYGPCGR.A
21	Aspartic protease inhibitor 9	gi|20137279	212	5.7/6.5	24.2/20.7	5	34	R.CPEDQFCAK.V<br>K.LCVSYTIWK.V<br>R.YNSDVGPSGTPVR.F<br>-.ESPLPKPVLDTNGK.E<br>R.TMLLETGGTIGQADNSYFK.I
22	Pentapeptide repeat protein	gi|53782287	292	5.1/5.7	14.5/22.1	5	35	K.GGGPYGAGVTR.G<br>R.GADFSLANVTK.V<br>K.VNLSNANLEGALTTGNTSFK.G<br>K.GSVIQGADFTDVPLR.E<br>K.FADGVNTVTGNATR.E
23	FBP aldolase Ia	gi|39816882	281	6.3/8.6	37.0/30.4	5	19	R.ALQNTCLK.T<br>K.AAQDTLLVR.A<br>R.SAAYYQQGAR.F<br>K.YTGEGESDEAK.K<br>K.ATPQQVADYTLSLLR.Q

1 From the NCBInr database.
2 Experimental p*I* and M*r* values were estimated using broad-range molecular standards (Bio-Rad) and the ImageMaster 2D Elite program (GE Healthcare).
3 Calculated p*I* and M*r* values were obtained with the immature sequences, using the Expasy 'PeptideMass' algorithm (http://ca.expasy.org/tools/pi_tool.htm).
